# Dynamics of Implementation and Maintenance of Organizational Health Interventions

**DOI:** 10.3390/ijerph14080917

**Published:** 2017-08-15

**Authors:** Mohammad S. Jalali, Hazhir Rahmandad, Sally Lawrence Bullock, Alice Ammerman

**Affiliations:** 1 Sloan School of Management, Massachusetts Institute of Technology, Cambridge, MA 02142, USA; hazhir@mit.edu; 2 Department of Nutrition, Gillings School of Global Public Health, UNC Center for Health Promotion and Disease Prevention (a CDC Prevention Research Center), University of North Carolina at Chapel Hill, Chapel Hill, NC 27599, USA; sallylb@live.unc.edu (S.L.B.); alice_ammerman@unc.edu (A.A.)

**Keywords:** implementation, adoption, health interventions, community prevention, obesity prevention interventions, system dynamics, systems thinking, endogenous dynamics, qualitative modeling, case studies

## Abstract

In this study, we present case studies to explore the dynamics of implementation and maintenance of health interventions. We analyze how specific interventions are built and eroded, how the building and erosion mechanisms are interconnected, and why we can see significantly different erosion rates across otherwise similar organizations. We use multiple comparative obesity prevention case studies to provide empirical information on the mechanisms of interest, and use qualitative systems modeling to integrate our evolving understanding into an internally consistent and transparent theory of the phenomenon. Our preliminary results identify reinforcing feedback mechanisms, including design of organizational processes, motivation of stakeholders, and communication among stakeholders, which influence implementation and maintenance of intervention components. Over time, these feedback mechanisms may drive a wedge between otherwise similar organizations, leading to distinct configurations of implementation and maintenance processes.

## 1. Introduction

One of the biggest health challenges in the U.S. is obesity: two-thirds of adults and one-third of children are overweight or obese [1,2]. Despite extensive public health efforts to control and reduce obesity, it still remains a critical crisis in the U.S. One of the main efforts has been the development and implementation of obesity prevention interventions in local communities and businesses [3]. These organizational interventions are often successful in the short term; however, their sustainability over the long term has been questioned. There are three distinct reasons for the importance and complexity of understanding the sustainability of obesity interventions:

- **Health consequences of obesity**: Excess weight is associated with many leading causes of morbidity and mortality, including increased risk of type 2 diabetes, hypertension, stroke, arthritis, and certain cancers, among others [4,5].
- **Economic impact of obesity**: The obesity epidemic has a potential economic impact in the U.S. Overall health expenditures in the U.S. from 2009 to 2012 were 17.7% of the GDP, the highest rate among 221 countries and more than twice the average for all countries (6.9%) [6]. The economic impacts associated with the obesity epidemic include medical, productivity, transportation, and human capital costs, which makes obesity-linked costs a noticeable portion of total national health expenditures [7,8].
- **Complexity of organizational health interventions**: Social systems are complex and implementing health related interventions within organizations is specifically complex [9,10]. Such interventions require changes in work processes that are often in a complex zone where uncertainty and lack of agreement are common [11]. We particularly select interventions involving multiple stakeholders, and this selection further increases the organizational complexity of implementation and maintenance of interventions.

Given the motivations of the study, we develop a causal loop diagram, building on organizational processes from case study evidence, to study the dynamics of implementation and maintenance of obesity interventions. We particularly focus on: (1) endogenous dynamics of implementation and maintenance of obesity interventions; (2) organizational interventions with multiple stakeholders; and (3) trade-offs in building alternative resources within each organization. They are further discussed below.

- **Endogenous dynamics**: The selected intervention programs provide health organizations with capabilities that have the potential to tackle obesity in a target population and provide additional benefits. A general belief is that the level of these capabilities (e.g., capabilities achieved by installing an outdoor playground in a child daycare center) is degraded over time and irrespective of other factors, but recent theories in the strategic management literature present the idea that such erosion could also be a result of systematic and endogenous dynamics within the organization [12]. These theories contend that, in addition to exogenous factors, capabilities can erode based on endogenous dynamics, which can take an organization from efficient to inferior capabilities. These endogenous dynamics could result from unfavorable temporal trade-offs between performance and robustness and long delays between the “better” and “worse” parts of temporal trade-offs [12].
- **Multiple stakeholders**: Health organizations often have multiple stakeholders [13,14,15], making it difficult to trace the shortcomings of dynamics of implementation and maintenance of obesity interventions and tease out the sources of those shortcomings. Multiple stakeholders not only have different goals and perceptions, but their goals and perceptions may also change dynamically over time [16]. The organizational sciences literature also shows that there is often no agreement in perceptions of success factors among stakeholders (e.g., see Davis [17]). In addition, research shows that the allocation of clear stakeholder responsibilities is often problematic (e.g., [18]). We contend that even if stakeholder roles and responsibilities are clearly defined in the development of interventions, other pitfalls in organizational processes driven by endogenous dynamics may turn cooperative or complementary interactions into conflicting interactions, which can potentially cause the erosion of intervention components. The organizational literature also stresses the importance of interactions and communication among stakeholders, but without an understanding of the underlying dynamics of such interactions, it would be hard to assess the consequences of insufficient interactions. In fact, the literature shows that even simple organizational systems, if they include time delays and multiple feedback relationships, can create complicated outcomes, which therefore become hard to anticipate via intuition [19].
- **Trade-offs in building alternative resources**: There are usually trade-offs in building alternative resources that increase the complexity of managers’ decisions for allocating effort to those resource investment [20]. For example, organizations are susceptible to focusing on doing what they know best and ignoring emerging opportunities [21,22]. They also routinely undervalue investments with long-term payoffs [23,24]. Empirical studies provide strong support for many quality and process improvement programs [25,26]. However, organizations often fail to fully realize these benefits because: resources are withdrawn from programs before complete results are observed; initial enthusiasm overwhelms the training capacity for keeping the programs effective; or seeking short-term gains overloads the system with demand and the organizations are pushed into a firefighting mode of operation [27,28,29,30]. Similar to process improvement initiatives, many organizational health interventions may be beneficial over the long haul, but require initial investments and delays before the benefits materialize.

The rest of this article is organized as follows. Study design and empirical setting is discussed in Section 2; Section 3 discusses data collection and research methods; Section 4 presents modeling, including the main mechanisms of the model; and Section 5 explains the heterogeneities across the case studies based on the endogenous dynamics in the developed model. The discussion is presented in Section 6.

## 2. Study Design and Empirical Setting

Studying the microfoundations and sources of variations in implementation and erosion of interventions calls for a few design characteristics. First, a focus on similar interventions across similar organizations is needed to control for possible alternative explanations. Second, observing various rates of erosion of intervention components across these cases may better elucidate how the underlying mechanisms vary across organizations. Finally, heterogeneity among the organizations under study would increase the generalizability of the results.

We used a polar case study design with three pairs of organizations. Each pair includes two similar organizations and the same intervention. In one of the two cases in each pair, the organization has been successful in implementing and maintaining the intervention; in the other, the organization has been less successful in sustaining the intervention. The three pairs of organizations vary in size and complexity which helps increase the generalizability of this study—complexity is highest in hospitals, moderate in child daycare centers, and lowest in food carry-out restaurants (see below for more information on each organization).

Moreover, we focus on well-defined interventions that require coordination among different stakeholders for their implementation, a common feature of most complex organizational processes. Additional comparability was achieved by focusing on interventions that are all related to health outcomes at the organization and community levels.

### 2.1. Shape North Carolina (Shape NC)

The first pair of cases looks at the Shape North Carolina (Shape NC) program, an initiative to introduce healthier food and more physical activity into child daycare organizations in the state. Changes in food provision, physical layout, and activity planning for children are designed in collaboration with Shape NC partners and provide the participating child daycare centers with improved market position, parental support, and local government support [31]. The intervention brings together previously developed programs in the state and integrates them with new research-based models. There are several major stakeholders involved in the Shape NC project, including Smart Start and Blue Shield of NC Foundation, NC Partnership for Children, Blue Cross, and researchers from UNC-Chapel Hill. The implementation approach aims to be both top-down and bottom-up. It is a community-based program and local experts in each community (at the county level) provide technical assistance in adopting, implementing, and maintaining the intervention. After multiple meetings with the project managers, we selected two child daycare centers from two counties with different levels of success in implementation and maintenance of the intervention—we call the successful center S1 and the less successful one S2.

### 2.2. Healthy Food Environments in Hospitals (HFEH)

Larger organizations are targeted in the second pair of cases, where hospitals partner up with North Carolina Prevention Partners (NCPP) in the project Healthy Food Environments in Hospitals (HFEH). The NCPP is a nonprofit that focuses on implementing healthier cafeteria food, more physical activity, and smoking policies in large organizations, among others. The implementation of these changes requires adjusting various vendors and organizational regulations, and introducing new layouts and incentives for various participants [32]. In return, the hospitals expect reductions in employee health costs, improved morale, and better experiences for patients, which all contribute to long-term competitiveness. The HFEH initiative conducts assessments for food establishments such as restaurants and cafés in hospitals and issues “Apple” certificates representing healthy organizations. There are three different Apple certificates: Red (indicating that the place provides “healthy and delicious” foods), Yellow (working towards Red), and Green (getting started). Particularly for hospitals, it is an excellence award showing that they provide healthy food choices and a healthy environment, not only to employees but also to patients and visitors. For this project, we selected two hospitals. Hospital one (N1), with over 600 beds, is a nonprofit general hospital, recognized as one of the top 50 hospitals in the U.S. It was the first hospital in the state to achieve the Red Apple. Hospital two (N2), with over 50 beds, is also a nonprofit general hospital with no Apple certificate.

### 2.3. Baltimore Healthy Carry-Outs (BHC)

The third pair of cases comes from the adoption and maintenance of the Baltimore Healthy Carry-Outs (BHC) initiative [33]. This initiative provided a random sample of small food carry-out vendors in a poor Baltimore neighborhood with assistance and incentives to implement healthier menu options and eating opportunities for the local community. The carry-out vendors were interested in this initiative because it distinguished them from the competition and also benefited the local community. Researchers from Johns Hopkins Bloomberg School of Public Health worked with the carry-out vendors to design and implement the intervention, which included changes in menu items, raw material suppliers, marketing and presentation of stores, and pricing of items. We do not present the BHC here—it is thoroughly discussed in [34,35]. Our discussions and analyses in this article are based on Shape NC and HFEH and are consistent with the BHC case, increasing the generalizability of our findings.

While the cases vary significantly in the size of the organizations involved, they share a focus on interventions that require collaboration among internal and external stakeholders and focus on processes that enhance health outcomes. These similarities allow us to compare and contrast the processes of implementation and erosion of intervention components.

## 3. Data and Methods

We selected the cases in consultation with the external stakeholders involved in implementing these interventions (i.e., the Blue Cross and Blue Shield of NC Foundation and the NC Partnership for Children, Inc. on Shape NC; the nonprofit NC Prevention Partners on HFEH; and Johns Hopkins researchers working on BHC; from here on, we will call these external stakeholders). Cases were selected such that enough time had passed since inception of the programs to allow for observation of erosion mechanisms in action. In each case, we conducted interviews (mostly in person and a few on the phone) with the main stakeholders involved in the implementation and day-to-day enactment of the intervention components. Interviews focused on understanding the components, how they were adopted and implemented, the parts that had been institutionalized, and challenges in maintaining them. Where available, archival data on the history of the cases were used to augment the interviews. Table 1 provides a summary of the interviews conducted to date.

Interviews for Shape NC and HFEH were conducted by the author and a joint researcher (PhD student) from the University of North Carolina at Chapel Hill. Interviews for BHC were conducted by the author. The research protocol was fully approved by the Virginia Tech Institutional Review Board (IRB number: 11-947). Interviewees included: (1) Interventionists as external stakeholders who designed and implemented the interventions; and (2) internal stakeholders engaged in the implementation of the interventions—center owners, CEOs, or department directors and their key staff for Shape NC and HFEH, and store owners for BHC. All interviewees were informed of the purpose and procedures of the research, and assured that the information would be confidential. They signed a consent form and received compensation of $35 per hour of interview for their time—some of them were not able to accept the money. A spreadsheet tracked interview information, including name, gender, ethnicity, and organizational role of interviewees, as well as date, duration, and location of the interviews. Interviews were recorded and transcribed into text. All transcriptions were then saved in MAXQDA 11 (VERBI Software–Consult-Sozialforschung GmbH, Berlin, Germany) for qualitative data analysis.

Data analysis began with coding the interviews for common themes related to implementation and maintenance of the interventions [36], following standards for qualitative research [37]. Coding of interviews was conducted by the two authors; any disagreements or concerns about the extracted data were discussed until consensus was reached. Coding helped in learning the mechanisms of implementation and maintenance through identifying key variables and relationships among the variables. For example, “financial benefits” (earned from implemented components) and “motivation of internal stakeholders to implement” are two variables extracted from the interviews, and the relationship between these two variables was that “financial benefits” had a positive effect on “motivation of internal stakeholders to implement”. More variables and mechanisms that have an impact on implementation and maintenance are discussed in the following sections. The emerging relationships among the extracted variables were then integrated into an evolving causal loop diagram [38]. The resulting causal loop diagrams embedded the key relevant mechanisms important for understanding how the interventions were implemented and how they eroded.

## 4. Modeling

**Figure 1:**
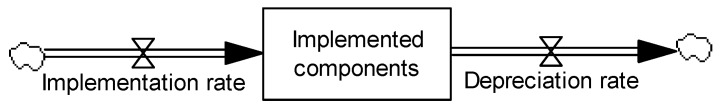
Basic stock and flow structure of implemented components.

We used system dynamics modeling to develop the causal loop diagram based on interview data. Causal loop diagram is a potential tool to understand the complexity of a system—increasingly used in public health in general and obesity literature in particular [39,40,41,42,43,44,45,46,47,48,49]. Similar to any project, an intervention includes several components that need to be implemented. However, not all implemented components are sustainable, and they may deteriorate over time. We assume that the intervention components are effective, in the sense that if properly implemented and maintained, they have a positive health impact. Therefore, it is the role of adoption, implementation and maintenance to make the intervention successful and sustainable. Stock and flow variables are key tools in system dynamics to present this mechanism. A stock presents accumulations (e.g., the number of implemented components). A flow presents the rate at which the stock changes (e.g., implementation rate is an “in-flow” and depreciation rate is an “out-flow” for the stock of implemented components). Figure 1 simply shows the basic stock and flow of the implemented components.

Next, we review the interview codes and capture the dynamic mechanisms affecting both the inflow and outflow of the stock, implementation rate and depreciation rate. It should be noted that some of the mechanisms (e.g., resources and motivation) are more complex than the others (e.g., self-funding) which required us to present more interview quotes for elaborating the more complex mechanisms and constructing the model.

In this section on modeling, most examples come from successful cases. In the following section on analysis, less successful cases are discussed more.

### 4.1. Resources and Motivation

Sufficient resources and motivated stakeholders are two necessary factors for implementation. The effects of resources and motivation on implementation are discussed as follows—examples are presented from each case study.

#### 4.1.1. Shape NC

Implementation of Shape NC is expensive. For example, building an outdoor playground requires financial resources. Both Shape NC centers (successful case S1 and less successful case S2) received the same initial grant to implement the intervention ($3000). The initial grant was crucial and helped the centers involved in the project. The grant was not enough to support the implementation of all components, but it helped the centers get started. One of the staff at S1 elaborates:

Financially, the first grant was $3000 which helped us get started. We won't have been able to get started if we didn't have that little push.

Another necessary resource for implementation is interventionists who help the centers implement the intervention. Without the effort and knowledge of the interventionists, the implementation would not be feasible. We consider financial resources and the efforts of interventionists to be key resources needed for implementation.

Resources are essential to implementing the intervention components; however, motivation is another needed element, without which ample resources are not of much help in kicking off and continuing the implementation. In Shape NC, competition was a key motivator for the center owners to join the project. The director of S1 discusses:

Forever we have always tried to get a leg up on other centers, because we felt like in order to get the children, we needed to be something a little different.

One of the staff at the local hub, an external stakeholder, says of the director of S1:

Sometimes it comes from—not because she knew about it but because she came to a meeting and someone else said this is what we are doing. So then she'll go out and figure out how to get that done in her center, so it is kind of that competitive. Her competitiveness is motivating her to do more… She is competitive. That is the first thing that comes to my mind when I think of Ms. [A.]. She was lower stars and she didn't care about increasing her stars [an assessment measure for the centers], because she knew she already had quality, she didn't care about the star rating system, but when that NC pre-k program came, she was like, what!? And sure enough she got it together. They had to apply to be the model early learning site—she made sure she had every piece that had to be in it. So she competed with 5 or 6 centers that applied and she made sure she had everything above what they could do so she could be that model learning center. So that competitive nature.

Furthermore, leadership support and involvement in the project is key to facilitating implementation. The level of support of organizational leaders goes back to their motivation. If leaders do not see the intervention as impactful, not only might they not support the project, but they even might be against it. This dynamic mechanism is already captured in the effect of motivation on implementation. We observed two completely different approaches to leadership support at S1 and S2. The director of S1 runs the child daycare center as a business center, so she potentially cares about competition with other centers in the community. In contrast, S2 was a center owned by a church and competition was not a big factor in motivation there. These different approaches by the leaders define the different initial motivation level of internal stakeholders.

#### 4.1.2. Healthy Food Environments in Hospitals (HFEH)

Interventionists from HFEH were also the key agents in helping the hospitals implement the intervention. One of the staff at the successful hospital (N1) elaborates:

I think they [interventionists] have been pretty helpful. There are a lot of things that we’ve done on our own. But we’ve used them as just an extra piece. I think we will continue to use it a lot more, because I know they built the toolkit, they’ve built a lot more resources, they have lots of webinars that they provide, and I think just continuing to communicate that to the rest of our staff internally so they know it’s there. I think it’s going to be helpful, because you don’t have to reinvent the wheel every time.

In addition to the efforts of interventionists, competitive advantage served as a driver of motivation in the HFEH intervention as well. One of the staff at hospital N1 mentions:

…we all felt it was the right thing to do. And I think quite frankly you don’t want to be the hospital that is not on the map, because they have a map of North Carolina that shows the hospitals that are and that aren’t [involved in the wellness program, the intervention]. I think if you were the leadership of the hospitals that aren’t, I think it might put some pressure on you to be the ones that are. Does that make sense? Because one of the things I learned about healthcare, I got into healthcare about ten year ago. I never realized really how competitive it is. It is very competitive. So you don’t want to be the hospital that’s in the market that doesn’t promote wellness because the one down the street is.

Along with competitive advantage, contribution to the health of community was a strong driver of motivation at HFEH; it was mentioned by several interviewees. One of the staff at N1 says:

…one thing that we are doing now more than in the early days, and this is part of our attempt or work to move from just the hospital, to expand from the hospital sector to other sectors. So now we are working to bring the program to whole communities. We look at the hospital to become an anchor for that community.

Another staff at N1 elaborates, particularly on community leadership:

I think we had a real commitment. As a community hospital, we very much want to represent to our community a healthy way of living, and we thought it is important to I guess be a mirror to our community. So it was important to our CEO, it was important to our wellness leadership that we partner with NC Prevention Partners to make a statement and to give us a pathway to becoming a healthier organization and being healthier for our customers.

The impact of leadership support was strong enough to distinguish the successful hospital N1 from the less successful hospital N2. We further discuss this difference in the analysis section (Section 5). One of the staff members at hospital N2 says:

…getting the directors on board with the staff and saying, ‘Hey, look, this is gonna launch. This is gonna benefit you.’ I think that’s the only way we can upscale it, because if you just email people, ‘Oh, this is happening, such and such.’ Okay, they most of the time just delete it...

One of the staff at the local hub of S2 believes that the commitment of directors is the factor that makes some child daycare centers more successful than others:

*…having a strong leader who is willing to do what it takes and be inspirational and motivating and facilitating into the roadblocks that they run into and of course staff who buys into that vision*.

**Figure 2:**
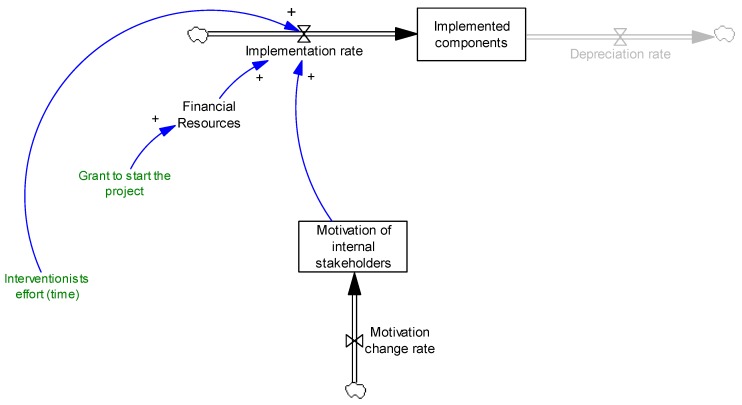
Effects of motivation and resources on implementation.

Therefore, the two key factors affecting implementation are resources and motivation. As discussed earlier, we consider resources to be interventionists’ efforts and financial resources. We also particularly focus on motivation, capture it as a stock variable, and study possible mechanisms that change it. Figure 2 presents the effects of motivation and resources on implementation.

It should be noted that we focus on motivation of internal stakeholders and assume that external stakeholders stay motivated. In fact, we observed highly motivated interventionists. However, we acknowledge that in other settings external stakeholders might become less motivated about the intervention over time. For the sake of simplicity, we only discuss the motivation of internal stakeholders in the model.

### 4.2. Communication and Design Quality

To keep the internal stakeholders motivated, a proper level of communication is needed between them and the interventionists. It helps not only to build trust among the stakeholders but also to address some of the issues in the design and adoption of the intervention—the design process requires sufficient communication among the stakeholders. Another important component that affects the quality of design and adoption of the intervention is the quality of effort of interventionists. If sufficient resources are available and internal stakeholders are excited about the program, yet the efforts of interventionists are of poor quality, the implementation process will face potential challenges. Thus, communication among stakeholders and the quality of effort of interventionists affect design quality. Examples to support these mechanisms are presented below.

#### 4.2.1. Shape NC

One of the staff at S1 mentions:

*It [our relationship with the technical assistants—interventionists] has been like a glove, we work very closely together. A lot of times they push me, because sometimes I get busy doing other things and [Ms.] R. [the key interventionist] gets me back on track; we should be doing this, change this,* etc. *She has been very instrumental with that and probably one of the key components to the whole program being successful is the partnership office.*

As discussed earlier, communication also helps improve implementation by reducing errors and facilitating implementation processes. Staff and interventionists at both centers highlight the need for sufficient communication. The director of center S1 elaborates:

Of course [Ms.] R. [the key interventionist] is phenomenal; she's worth her weight in gold! She has come out and sits down with me for a few minutes and I am thinking, you know I can't go this next step. I just really don't want to go out and beg for more money or more help. She will say, Ms. A., you just have to... By the time she gets through, I'm thinking this is going to be a piece of cake! I go do whatever we need to do and I don't always make the best decisions with the people that we hire, but we look pretty good out there and kids love it. That is name of the game.

In a more explicit example, one of the interventionists of S1 explains a design issue in serving healthy meals:

Actually really the biggest killers are the teachers. If they say, eww... I'm not going to try that. Then the kids react the same… The little bit of stuff you hear from the kids is the food, but mostly it’s because they heard a teacher say they didn't want to eat something. If you get the teachers on board and get them to introduce it and be excited about it and have taste testing parties.

This design issue was raised and solved through the communication between the interventionists and internal stakeholders—training sessions for teachers were accordingly planned. Moreover, quality of efforts of interventionists affects the adoption and later the implementation of the intervention. If the quality level is low, more problems are encountered later in the intervention implementation process. Quality is rarely perfect, so the implementation of some intervention components can often be problematic. In Shape NC, technical assistants (TAs) who were the key interventionists directly in touch with the center owners and staff were highly trained, so we can expect that the quality of their efforts was at a good level. One of the staff at the local hub mentions:

…in the beginning, we really focused on the working and training and as the hours grew I think we put a little more of a hands off rule specially this last year, because I think they [TAs] feel more confident in their field compared to the previous years and they had a lot more experience behind them.

Another staff member also elaborates:

They [the TAs] have learned to believe in the program so strongly... I've done this training for every employee that I've had.

In addition, the culture of sharing is noticeable among the interventionists, which helps improve the quality of their efforts. Another staff member at the local hub says:

Everyone is very willing to share resources. If you need something that you don't know, you can just email them and if they don't have it they will find it. So, I think we have a good system for sharing resources and I try to come back and share it with all of the TA girls, so it can spread throughout the county.

#### 4.2.2. HFEH

One of the staff at hospital N1 elaborates:

…they [interventionists] are great at answering as soon we have questions. I think we get an e-mail every week or two with maybe an upcoming webinar or anything that may be of interest to us. So, I’d say maybe once a week, once every two weeks we’re in contact with them… We communicate a little more often around the times that we take the assessments, because we’re gathering information, preparing slides and getting things ready to show that we’ve met certain requirements to earn an A in those areas.

Given the large organizational size of HFEH (i.e., hospital size) compared with Shape NC, there was a higher demand for communication among the internal stakeholders. One of the staff at N1 explains:

We have the e-mail blasts that go out every week, weekly reminding people that you have the opportunity to earn points, don’t forget to go in and track your exercise, bulletin boards, [and] staff meetings.

One of the interventionists at N2 explains how they communicated with their upper level stakeholders—state-level stakeholders who funded the program—in the design of the intervention.

When we were first designing the intervention, we’ve always had a very open, qualitative approach, where we read the science, we write it down, but then we spend a lot of time with our stakeholders, saying, really, what do you need? Like what are your stresses, what are your frustrations, what are your pain points? And then put the two together, so that it’s a little bit more user-friendly, and it really meets their interests, instead of just our goals.

However, we observed that there was not much effective communication between the interventionists and internal stakeholders at hospital N2. This lack of communication can potentially reduce the quality of intervention component, which later reduces the motivation of internal stakeholders.

We also observed several design issues mostly for physical activity components at hospital N2. One of the staff members says:

I think for our program here, you can either participate here at the gym, or you can do it at home and be part of the wellness program… You don’t have to be linked in here, but that seems like the biggest issue. Concern-wise, I think, the few people that I’ve mentioned, like insurance benefits and stuff like that [incentives for the wellness program]… [but] I think some people feel like it kind of steps into their personal lives too much.

Another staff member also adds:

I think the biggest challenge I could see… as far as I enjoy exercising, but the biggest challenge for me, and motivational factor for me is the transition with the weather or the seasons to still keep people motivated... So you just incorporate it into your daily routine, [but] I could see [it] as a really big challenge, because they drew people in at the first part of the year, but then with the warm weather, it’s kind of like, how do you keep them engaged? So I think that’s been one piece that hasn’t quite been figured out yet.

These two design issues were not discussed with the interventionists and they remained unsolved. The director of the wellness program at N2 mentions another design issue in a physical activity component, which was raised and discussed with the interventionists and they could plan for other alternatives:

Like the first year, we started something called Walking Wednesdays, which was supposed to be, the idea was, that every Wednesday employees would gather and walk during their break time. Complete flop...! You know, it became too difficult to coordinate that sort of things, so we pretty quickly found out. This is not effective; this is not a good use of time for our staff. Let’s pull back and put in something else that’ll work a little bit better.

He further explains how these design issues, along with an issue in the design of incentives for the wellness program, can reduce the motivation of internal stakeholders and hospital staff members:

I think some of the wellness challenges, as I mentioned, have been a little bit flops. Not a little bit, they’ve been flops! I do think some different incentives will be a big help, even if they’re not directly tied to insurance premiums, if we made the incentives a little more relatable to insurance cost, I think that would be a big step in the right direction as far as incentives go. The incentives we have now just frankly do not motivate everybody. They’ll only be motivators for some people, which I guess is true of any incentive, but I think having some more incentives will just give a broader spectrum of people to incentivize or to motivate.

**Figure 3:**
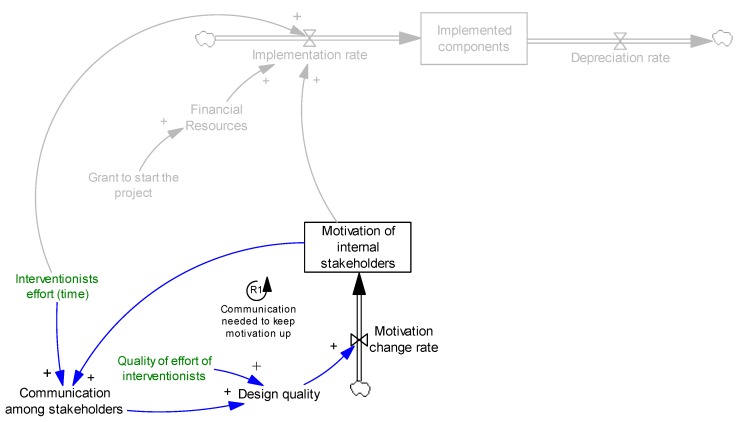
Effect of communication on motivation. R1 presents a reinforcing feedback loop.

It should be noted that to keep the communication at a desirable level, stakeholders need to be motivated enough to communicate, otherwise communication decreases. This completes loop R1, Figure 3. A feedback loop is called reinforcing (shown as R# in figures) if an increase (decrease) in any variable in the loop results in further increases (decreases) when we trace the causal links across the loop back to the original variable.

### 4.3. Stakeholder Alignment

#### 4.3.1. Shape NC

Stakeholder alignment is another component that helps reduce errors in the adoption and implementation of the intervention. A staff member at the local hub elaborates:

The owner may say they want it but the director may not be fulfilling the extent of the intentions made. And the owners aren’t in the loop; it is the directors [who] are in charge. The directors are the ones on [the] go but the owners are the ones who can put a brake on the project. The owner may switch the bandwagon—we have seen that a lot.

Another staff member at the local hub explains:

I think motivation was high but it dipped when it came to how to implement the project, because they weren’t quite sure what was going on and what happened. So, potentially, I think the motivation varied but now everybody seems pretty motivated. They were pretty jazzed and excited and had really good positive stories to share. I think motivation is back up top.

**Figure 4:**
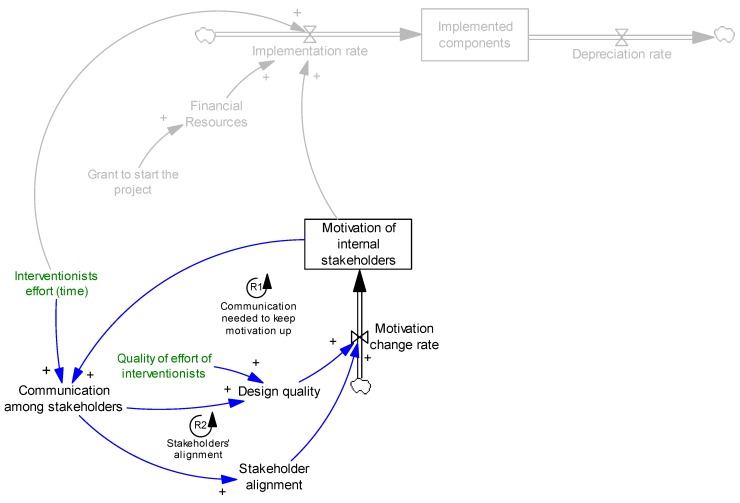
Effect of communication on stakeholder alignment.

Once the motivation dipped down because of conflict among stakeholders, communication helped them raise and deal with the issues. Hence, communication among stakeholders increases stakeholder alignment, which eventually results in increased motivation of internal stakeholders. This mechanism is presented in loop R2, Figure 4.

#### 4.3.2. HFEH

In HFEH, stakeholder alignment was not a major issue—there were some differences in perceptions and intentions of the wellness program director and the CEO at hospital N2, and we discuss this in more detail in Section 5.

### 4.4. Effects of Costs and Benefits on Motivation

As a result of the intervention implementation, internal stakeholders might observe new costs and benefits. A major benefit for the organization was competitive advantage as well as having an impact on the health of the community. There were also major costs of implementation of the intervention components, such as installing an outdoor playground in Shape NC.

#### 4.4.1. Shape NC

The director of S1 discusses this:

I would like to do what [Ms.] R. [the key interventionist] has suggested. It seems like we'll have a cook for a while and then they are gone, but what I'd really like to do is have a tasting on a Friday afternoon and do some new recipe and let parents have a taste. Have parents come and taste the new recipes and ask them if they think their kid would like it and give them the recipe to make at home… That would be good advertisement.

#### 4.4.2. HFEH

The implementation of the intervention imposed significant costs on the hospitals. Not only was the implementation costly, but also the projection of the consequences of some of the components did not seem beneficial to some staff. The program director at hospital N1 elaborates:

Everybody told me we were going to lose money, that the sales were going to hurt, because people want French fries; I said don’t worry about that. We actually increased revenue. It’s been pretty good…. We looked at it after six months and we were up about 18% on our growth. Overall since we took over [(took out the fryers)] five years ago we’ve been up to around $2,000,000 revenue of the year. And that’s just [for] serving better food, and brought in a whole new customer base. If you looked at the snapshot of the customers who were eating six years ago, it was heavy environmental services maintenance guys who want fried food, fried chicken, that kind of crowd. And when we introduced healthier food, we started seeing more doctors, more nurses, [and] more outside people who were eating, because it was a healthy way to go. So we brought in a new customer-base by adding healthier foods.

Another staff member at hospital N1 mentions:

We did a whole renovation, and we were going to invest that money to get rid of the fried food, we certainly needed to be able to support that. [When] it comes down to money, you don’t want to do something that will really hurt your business and you’re just left hanging out there if this wasn’t a good idea. And we wanted to make sure that the idea we were doing was a good idea, both financially and nutritionally and all those things.

**Figure 5:**
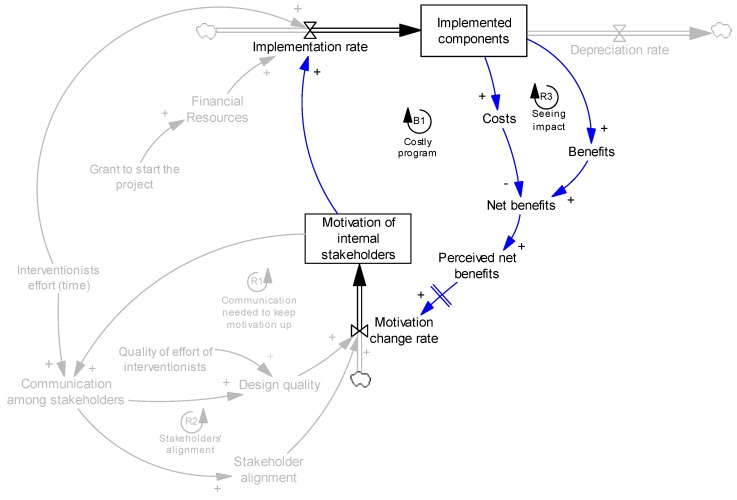
Effects of costs and benefits on motivation. B1 presents a balancing feedback loop, where a perturbation in one variable is attenuated once we trace its impact across the loop back to the original variable.

Therefore, the internal stakeholders compare the costs and benefits (we added the variable “net benefits” in the model, net benefits = benefits − costs), and if they observe more benefits than costs (when net benefit is positive and loop R3 dominates loop B1 in Figure 5), they will be more motivated and consequently will collaborate in implementing remaining components and maintaining those previously implemented. However, this mechanism is dynamic and might change over time. For example, if implementation gets more and more expensive such that the costs are not worth the outcomes, the perception of the owners tends towards being against the program, making them less motivated to contribute. It should also be noted that the perception of the net benefits does not change motivation immediately—this delay itself can be another complexity in the model. Figure 5 presents these mechanisms:

### 4.5. Self-Funding

As already discussed, implementation is costly, and both child daycare centers and hospitals need sufficient financial resources to move forward and implement the intervention components. This requires that internal stakeholders invest in the intervention and self-fund the implementation—in addition to grants from external stakeholders, if any.

#### 4.5.1. Shape NC

By seeing the impact of the intervention, center owners become more motivated to implement, and may be willing to provide financial resources if the initial grant does not cover all implementation costs. The director of S1 elaborates:

A lot more money should be put into this than what is being put in now. I've always liked to break new ground, which is what I've done. But I've spent way more money than I received, but it has been well worth it to this point… I've put my own personal funds in and move money to this out of the budget. Not everybody can do that. But the more you put into a program the more you get out of it. They also need checks and balances—you need to make sure that the money that you are putting in is really doing what it needs to do. In some instances it is not, and that is a waste… I would say that I have spent probably three times the amount of my own money of the scholarship that we've received.

One of the staff at S1 adds:

Ms. A. [the director] went way above and beyond that as far as spending. She built a well just to water the plants—it takes away from her water bill, but by the time you figure out how much she spent on that vs. the cost of the well, it was probably no comparison. But it is out there and it is wonderful. It is great the kids can turn it on and we don't get excited if the water is running a little longer than it should. They've learned to water their plants. So that little bit of a financial thing [initial grant] was like the carrot out there. Just kind of got us started. By no means did it support everything that we did.

#### 4.5.2. HFEH

The initial grant at Shape NC helped the centers get started, but HFEH did not offer any financial support to the hospitals. This highlights the importance of the effect of motivation, such that if internal stakeholders, particularly hospital administrators, are not motivated enough to fund the project, implementation of the resource-based intervention components (such as renewing the hospital restaurant) may not be feasible. While motivated administration at N1 provided financial resources for the implementation of the intervention components, less motivated administration at N2 did not provide any financial support. The program director at N2 elaborates:

We really have had no resources to allocate. We don’t have a budget for wellness per se. Now, of course our department has a budget, but there never has been a particular amount set aside for employee wellness specifically. So all of the things that we have tried to do since the beginning have been low to really no cost movements.

**Figure 6:**
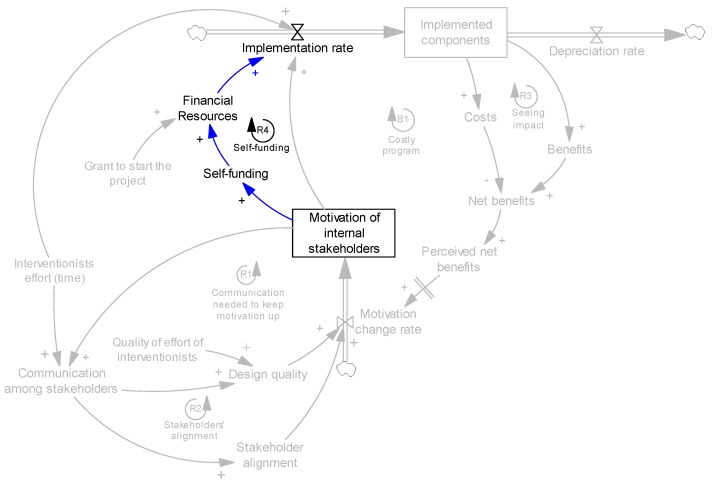
Self-funding mechanism.

The self-funding mechanism is presented in Figure 6, loop R4. It should be noted that, in all organizations we studied, the affordability of funding the implementation was not an issue. Here, we consider motivation as the potential, main driver to allocate financial resources and self-fund. Our endogenous perspective helps us analyze how variations in other variables of the model can change motivation (through the causal links) and consequently increase or decrease the willingness for self-funding.

### 4.6. Non-Dynamic Factors Affecting Motivation

**Figure 7:**
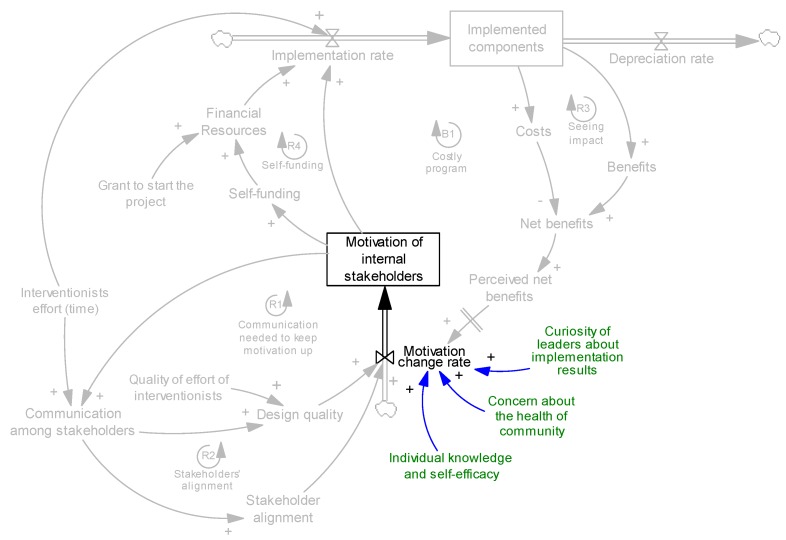
Examples of non-dynamic factors (green parameters) affecting motivation.

Motivation is also impacted by other factors, such as individual knowledge and beliefs about capability and self-efficacy for carrying out the new processes [50]. Other factors affecting the motivation of internal stakeholders could be novelty and the curiosity of leaders (owners/admins) about implementation results and their concern about the health of the community—examples presented for motivation (Section 4.1) and self-funding (Section 4.5) mechanisms support these two factors. While such characteristics have a potential impact on motivation, we do not include them in our analysis of endogenous mechanisms because they usually do not change dynamically during the evolution of an intervention. These parameters are added in Figure 7 (shown in green).

### 4.7. Depreciation and Maintenance

**Figure 8:**
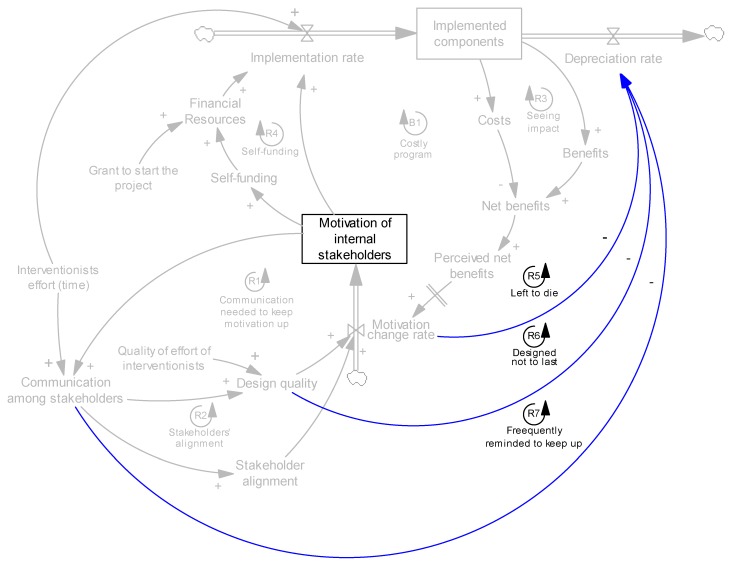
Effects of motivation, communication, and design quality on maintenance of intervention.

Up to this point, all the mechanisms presented affect the implementation of interventions. Intervention maintenance emerged as another critical factor. Implemented components erode when they deteriorate, depreciate, or are otherwise scrapped, and are not renewed—these processes continually reduce the number of implemented components. However, the depreciation rate is also endogenous, as it depends on other factors. Through the interview data from internal stakeholders and interventionists, we learned of three key factors: motivation, communication among stakeholders, and design problems. We find that motivated internal stakeholders are more likely to internalize and sustain changes without external prompts (R5, Figure 8). High-quality designs foresee and correct for the most common modes of failure and thus include lower baseline depreciation rates (R6, Figure 8). Finally, communication can help remind internal stakeholders of the need to sustain changes and fix emerging problems (loop R7, Figure 8).

#### 4.7.1. Shape NC

To keep the program successful, intervention components must not only be properly implemented but also maintained; otherwise, they will deteriorate over time. We already indicated that communication is needed to identify implementation errors and consequently results in more progress in implementation. Lack of communication not only makes implementation problematic, but also increases depreciation of those components already implemented. One of the interventionists says:

…the frustration and the motivation at the beginning and the lack of communication just sour it all and it never recovered. And when we select folks, there is this criterion but you have got centers, directors or owners who may not be that good at communicating.

In fact, communication can help remind internal stakeholders of the need to sustain changes and fix emerging problems until intervention components are fully institutionalized and transformed into organizational routines. Moreover, motivated owners are more likely to sustain the changes without external prompts, and the quality of implementation influences the baseline depreciation rates.

An example from S1 shows how a small design problem was about to deteriorate an intervention component, where the implementation of a garden for kids focused only on children and not on teachers. However, with more communication through training, the issue was resolved. One of the interventionist further elaborates the story:

Now with the garden, there's some enthusiasm, there's motivation from the teachers. Whereas some time back, I was not seeing much motivation from the teachers. After the training, now I'm seeing teachers like little bees running around outside with the kids. They are playing soccer and it is kind of weird how it happened. I saw a big shift when they opened up the fencing and allowed more space, more free spaces for kids and teachers to move.

#### 4.7.2. HFEH

The three mechanisms affecting the depreciation of implemented components were more noticeable in Shape NC than in HFEH. Interventionists in HFEH paid close attention to the maintenance while designing the intervention components. One of the key interventionists elaborates:

We’ve designed the program in order for them to easily maintain things over time, because one of the things that we encourage is that they continue to take the assessments to make sure that they’re maintaining that high level once they’ve achieved it.

Hospital N1 had not noticed as much depreciation by the time of the interviews, and hospital N2 was not able to implement many of the intervention components. The program director at N1 was fairly aware of the effect of motivation on maintenance. He elaborates:

[The main challenges to maintain the program is] just to keep people interested and excited. You want to do something that is different enough each year to keep them engaged, but you don’t want to change it so much that they go, ‘Uh, here we go again’, but something new, something completely different. We just learnt this one, now we’re starting something new.

One of the staff at N1 nicely summarizes the effect of motivation:

[To be successful] I think you’ve got to have buy-in in that. I think that goes back to the culture, but I think you need to understand why you’re doing this, what’s the benefit of doing it, in that you stick with it, dig your heals in the ground, this is it, this is what we’re doing, this is our program, and then eventually it will become a culture thing.

Another staff member at N1 answers the question, “Was there any part of the program which was not maintained well”:

Not really! Honestly, it’s just continued to grow bigger and bigger and bigger, and haven’t seen it backslide at all.

The preceding two examples support the hypothesis that once new practices, intervention components, are institutionalized and transformed into organizational routines, they will sustain and emerging problems will be fixed. The three factors affecting depreciation rate are presented in Figure 8.

## 5. Analysis

The focus in Section 4 was on the relationship between the key variables to develop the casual loop diagram. In this section, we explain how the dynamic mechanisms in the model and trade-offs in the endogenous mechanisms can distinguish successful cases (child daycare center S1 and hospital N1) from the less successful ones (child daycare center S2 and hospital N2).

### 5.1. Shape NC

In a nutshell, high motivation of internal stakeholders was the key to success at S1 because it encouraged original implementation and reduced future depreciation, allowing for sustainability and growth of the intervention and its financial benefits to materialize. Here we describe the mechanisms that helped increase and maintain the level of motivation of internal stakeholders. Expanding on examples discussed in Section 4.1, personal characteristics of the center owner (competitiveness and leadership interest) and situational factors (facility attractiveness and leadership role in the community) created a desirable initial level of motivation. Over the course of implementation, internal staff realized the impacts of the implemented components. In fact, comparing the perceived benefits (e.g., making center S1 the leader in the child daycare business in the community) with the costs of the intervention, their overall perception of the intervention was that it was a beneficial program (where loop R3 dominates loop B1 in the model, Figure 5). Therefore, the initial high motivation of the internal stakeholders, particularly the center owner, was maintained. With her and the staff motivated and excited about the program, they were willing to communicate with the interventionists, receive advice from them and solve possible issues throughout the implementation processes (loop R1, Figure 3).

Motivated internal stakeholders at S1 helped customize the intervention components, which facilitated further maintenance. This required more communication between the internal stakeholders and interventionists to fix the issues and plan for additional implementation of modifications, which eventually transformed the intervention components into organizational routines.

Once the intervention components were institutionalized, the internal stakeholders continued to maintain the intervention with or without the help of interventionists. A staff member at S1 mentions:

We re-hauled the entire playground, added the trike path, planted fruit trees and other trees, [and] had parent work days. We come out on Saturdays some days. Just want needs to be done, step by step. We've had about four work days where we built things out here without the children... We try to keep the staff motivated, because at first they didn't really get it, but now they are adding it to their lesson plans and thinking about it all the time. They picking books that have fruits and vegetables and fresh foods in them—farm books and things like that, instead of your typical fantasy princess stuff.

The initial motivation of internal stakeholders in center S2 was not as high as in center S1. If the center directors are not motivated enough about the intervention, they may affect the perceptions of the staff and eventually they will not commit to better implementation and maintenance of the implemented components. One of the staff at the local hub of S2 elaborates on the commitment of directors and how it can make some child daycare centers more successful than others:

…if you have a director X and center Y, and church you probably know this but [the] church based child care doesn’t have high requirements for profit and they give them a pass on certain things. So it’s different to see where the center director gets it and gets on board with it to where you have had a good relationship with the local partnership and they are prompt to do these kinds of things.

### 5.2. HFEH

Similar to child daycare center S1 in Shape NC, internal stakeholders at hospital N1 joined the project with high initial motivation. Their motivation was then maintained over time by communication with interventionists, fixing the issues, and seeing the impact of the intervention. In Section 4.1, we noted some examples of benefits to hospital from implementing the intervention, such as competitiveness and community leadership.

Consequently, with motivated internal stakeholders at N1 the intervention was maintained well and the internal stakeholders implemented further practices. Moreover, the motivation of internal stakeholders accompanied by hospital administrative support enhanced implementation and maintenance of the intervention. The motivation of administration is an essential factor, particularly in large, complex organizations like hospitals. For example, given that there was no initial grant to start the intervention, self-funding was a necessary factor to cover program costs. Without the support of hospital administration, funding would not have been secured.

Overall, in the HFEH project, external stakeholders were faced with lack of leadership support in several hospitals, so much so that they tried to get verbal confirmation of administration support for the program. One of the interventionists mentions:

Something that we require hospitals to do before they start working with us is to sign a CEO commitment form. That form basically says: ‘Yes, personally I support this but also I am going to put in my strategic plan, we’re going to work on this as an organization, and there are the people that want to work on it from my hospital.’ Having that leadership support is just so important, and as we go out and we visit hospitals and see what they’re doing on the ground, and seeing the CEO support, we definitely see those hospitals as moving forward more quickly than hospitals that have just mediocre or no support for the wellness program.

Chief executive officers or directors may have different leadership skills and strategies, yet their support is tied to their motivation and affected by endogenous organizational mechanisms, i.e., the feedback loops affecting motivation in the model. With motivated internal stakeholders along with the support of administration, the hospital practices intervention components and gradually such components integrate with organizational processes and are routinely maintained. The wellness program at hospital N1 experienced the transformation of new practices into organizational routines. One of the internal stakeholders elaborates:

The wellness program is just a part of our life here. People are used to it, they’re very committed to completing their preventive items, and that is still a part of the wellness program with vitality, there is a prevention component to that.

This transformation facilitates the maintenance so much that interventionists, along with internal stakeholders, believed that maintenance was not as hard as implementation. One of the internal stakeholders says:

I think it’s pretty easy to maintain [the program] once you get there. Getting there could be challenging for some people.

While high motivation reinforces several dynamic mechanisms in the model that lead to better implementation and eventually better maintenance of the intervention, low motivation can act in the opposite manner. Stakeholders who are not motivated might not communicate with the interventionists as often as needed, causing them to face more challenges along the way to implementation, which eventually decreases their motivation. Consequently, reduction in financial and leadership support for implementation results in not fully or properly implementing the intervention components. Internal stakeholders then perceive the program as a whole as not beneficial, and such negative perceptions of the program feeds back to their motivation and makes the situation even worse. This was the situation in hospital N2. The director of the intervention at hospital N2 answers to the question, “Why did the hospital decide to join the program at the beginning”:

I really don’t know! I’ve never been able to find out the actual answer. I think it was just because somebody had brought it up in a meeting, and the CEO at that time was like, ‘Okay!’ and didn’t really know anything about it, because I actually went back to my HR director… maybe two years ago, around the end of the first year of the program, when I had been trying to offer a lot of proposals for things to do, and was not really getting anywhere, and she said, I’m talking about the CEO, said, ‘he doesn’t care. He doesn’t know what’s going on. He doesn’t have any interest in it.’… She was being nice to me, telling me that information. She just said, ‘You know, you do with it what you feel like you need to do.’

Hospital N2 tries the best they can to save money, even by laying off employees. Employees are also so busy with their daily tasks that they do not have any additional time to spend, e.g., on attending wellness programs. In fact, since the implementation was not properly done, the intervention components never turned into organizational routines.

## 6. Conclusions

In this article, we used multiple comparative case studies and developed a causal loop diagram to study the dynamics of implementation and maintenance of organizational health interventions. In each case, the introduction of a new practice entails designing and implementing various components, such as physical components (e.g., playground) and incentives (e.g., for employees to exercise or quitting smoking). The design and implementation in each case can be seen as a project comprised of various components. Execution of these components informs the progress of the implementation phase and depends on the quality of interventionists’ efforts and the time they allocate to implementation, and, more importantly, the motivation of internal stakeholders to actively contribute to the project. Besides implementation, the maintenance of newly implemented components is key to the long-term value of the intervention: The new practices only have the potential for impacting organizational performance if they last. Our qualitative modeling work elucidates a few exogenous factors (such as quality of efforts of interventionists and the existence of program grants), as well as some endogenous mechanisms, that moderate implementation and erosion rates. Below we discuss our findings—focusing on the main endogenous mechanisms of motivation, communication, design quality, and their interconnections—for health professionals and researchers to increase the sustainability of intervention programs.

Based on several endogenous reinforcing mechanisms, we present that early differences in the implementation of interventions can end up with very different paths to success. For instance, initial high motivation of internal stakeholders makes the organization become more motivated to communicate with the interventionists, provide financial support for implementation, and institutionalize the new practices, and thus see lower costs for maintaining them, further increasing the perceived benefits, compared to another organization that started off on the wrong foot. In addition, for one organization, lack of initial motivation may limit the bandwidth of communication, reduce the quality of design, reduce the stakeholder alignment, and lead to much rework and wasted resources in the implementation phase, while another organization thrives. In addition to making implementation problematic, these early differences can also be amplified and lead to very different erosion rates. For instance, internal stakeholders who are less motivated will be less likely to communicate with the interventionists (e.g., to raise emerging issues) and hardly internalize and sustain changes without external prompts. Therefore, interventionists should monitor the motivation of stakeholders and include motivational components in the design of the intervention.

Furthermore, design quality emerged as an important aspect of the studied interventions because a well-designed intervention matches the requirements for the organization at hand and thus is less costly to organizations, may include more benefits, and is easier to implement and maintain (see examples in Section 4.2 and Section 4.7). The quality of design partially depends on the knowledge and skills of the designers (in our cases, the interventionists), but more importantly on the communication between designers and internal stakeholders. In fact, the customization of the intervention based on the characteristics of each organization requires ample communication between the interventionists and internal stakeholders. Communication between internal stakeholders and interventionists sets the tone for whether new practices are taken up and modified to best fit the organization’s internal and external environment, or are ignored or even actively resisted. Taken together, communication and design quality create potential reinforcing loops: Increased motivation facilitates better communication, which improves design, enhances perceived benefits, and keeps the internal stakeholders motivated. Interventionists often spend more time on the design of the intervention than on communication with stakeholders. However, they should allocate more time to communicate with other stakeholders as a small shortfall in communication sufficiency can have huge consequences on the overall sustainability of the intervention.

In all our cases, motivation of internal stakeholders emerged as a critical part of explaining performance heterogeneity across similar organizations. As discussed earlier, reinforcing loops can amplify differences between two programs, if one faces initial lack of leadership support that reduces motivation and communication and sows the seeds of future problems. Variations in the development of each intervention were observed, but much of the difference in longer-term performance levels could be better explained by the motivation of internal stakeholders. We present several endogenous mechanisms which change the motivation dynamically over the course of implementation, i.e., communication between internal stakeholders and interventionists, intervention design quality, stakeholder alignment, and impact of implemented components.

Our analysis points to a few reinforcing mechanisms, moderated by motivation, communication, and design quality, which impact both initial implementation and erosion of intervention components. We suggest that these reinforcing mechanisms can create path dependencies in capability evolution trajectories (capabilities achieved due to the implemented interventions) across organizations, leading to heterogeneity in performance, even when the elements of the intervention are relatively well-known. Similar dynamic mechanisms were presented for the BHC case in [34]. The basic design, implementation, and maintenance of new organizational processes are shared in developing many health interventions. In fact, there is much variability in the three interventions and organizational contexts explored here. Therefore, qualitatively, the dynamics discussed will be relevant to many settings. However, the quantitative analysis will be more dependent on the organizational context and indicates which loops will dominate the dynamics in which organizational settings.

Finally, the endogenous perspective we employed offers a distinct way of interpreting organizational performance and change. In this perspective, organizations may diverge into different performance trajectories, not because the actual payoff landscape is very rugged [51] and finding the best configuration is computationally intractable [52], but because actions taken by organizational members and results observed complement each other in endogenous feedback processes. While the strategic importance of some reinforcing processes, such as learning curves [53] and network effects [54], are well established, we think this explanatory engine can be fruitful in understanding a much wider set of phenomena in strategy, particularly in the health literature. The feedback processes among communication, motivation, and design are just a few examples. Using this perspective, researchers can identify and quantify the various feedback processes relevant to each health organization setting, and managers can seek to activate specific feedback loops in their favor and leverage those to distinguish their organization from the competition.

## Figures and Tables

**Table 1:** Summary of the interviews.

Organization	Interviewees	Number of Interviews	Interviews Length (min)
Healthy Food Environments in Hospitals (HFEH)	Interventionists and other stakeholders	5	400
	Internal stakeholders—Case 1 (N1) *	3	140
	Internal stakeholders—Case 2 (N2) **	6	170
Shape North Carolina (Shape NC)	Interventionists and other stakeholders	11	695
	Internal stakeholders—Case 1 (S1) *	8	230
	Internal stakeholders—Case 2 (S2) **	4	190
Baltimore Healthy Carry-outs (BHC)—presented in [34]	Interventionists and other stakeholders	5	225
	Internal stakeholders—Case 1	1	50
	Internal stakeholders—Case 2	1	60
Total		44	2160

* S1 and N1: successful cases; ** S2 and N2: less successful cases.
